# Antibody-based therapies for COVID-19: Can Europe move faster?

**DOI:** 10.1371/journal.pmed.1003127

**Published:** 2020-05-05

**Authors:** Michel D. Kazatchkine, Michel Goldman, Jean-Louis Vincent

**Affiliations:** 1 Global Health Center, Graduate Institute, Geneva, Switzerland; 2 I3h Institute, Université libre de Bruxelles, Brussels, Belgium; 3 Department of Intensive Care, Erasme University Hospital, Université Libre de Bruxelles, Brussels, Belgium

## Abstract

Michel Goldman and colleagues call on the European medical and scientific community to coordinate efforts on immunotherapy-based approaches to coronavirus.

Countries of the European Union (EU) are among the most seriously affected areas by COVID-19. Deaths have exceeded 75,000 across the EU within 2 months of the epidemic. The main cause of death is respiratory failure complicated, in a number of cases, by shock and multiple organ failure [1]. There are no therapeutic options beyond symptomatic intensive care treatment for severely ill patients at this time. We call on European physicians and scientists to urgently evaluate the potential of antibody-based therapies in this context.

Recovery from infection with SARS-CoV-2 is associated with the generation of neutralizing immunoglobulin G (IgG) antibodies to the virus within 6 to 10 days of symptomatic infection [2,3]. Studies in rhesus macaques indicate that monkeys infected with the new coronavirus produce neutralizing antibodies and resist further infection [4]. Although no data are available on the duration of acquired anti–COVID-19 immunity, studies of the SARS outbreak had shown that people infected with SARS coronavirus generated protective immunity that lasted for up to 10 years [5].

Immune convalescent individuals might contribute to the treatment of critically ill infected patients through donation of their plasma. Preliminary uncontrolled studies in China indeed indicate that infusion of convalescent plasma might benefit patients on mechanical ventilation [6]. On this basis, programs of convalescent plasma therapy (“passive immunotherapy”) have been launched in several centers in the United States [7]. Passive immunotherapy has been conceptualized over 100 years ago with the pioneering works of von Behring and Roux. Europe has played a major role in the development of passive immunotherapy with plasma and ensuing hyperimmune immunoglobulins, F(ab’)2 or Fab fragments of immunoglobulins and monoclonal antibodies. Effective neutralizing antibodies for passive immunotherapy have been generated in recent outbreaks of H5N1 [8,9], SARS [10], and Ebola [11].

Blood transfusion centers in Germany, France, Italy, the Netherlands, and Belgium are launching plasma collection campaigns focusing on donors screened for anti–COVID-19 antibodies. Anti–COVID-19–rich plasma will then be used in intensive care centers in the framework of controlled prospective clinical trials. Currently available assays will allow selection of plasma donors with high titers of neutralizing antibodies for plasma therapy and the preparation of hyperimmune intravenous immunoglobulin. Efforts are underway to harmonize protocols. However, further coordination is urgently needed to ensure that significant conclusions can be drawn to inform about the benefit or lack of benefit of this approach.

We call on the medical and scientific European community to urgently coordinate research efforts on passive immunotherapy, share data and protocols in real-time, and pool efforts to decrease as much as possible the time for validating immunotherapy with donated plasma. Analyzing and conciliating data, taking into account differences in existing protocols, could be done by a dedicated European task force. We call on the European commission, the Innovative Medicines Initiative (IMI), national research funding bodies agencies, and plasma fractionating plants to accelerate and coordinate the development of therapeutic immune globulins and laboratory-made immune components to rapidly substitute to donated plasma.

## Abbreviations

EU: European Union
IgG: immunoglobulin G
IMI: Innovative Medicines Initiative
